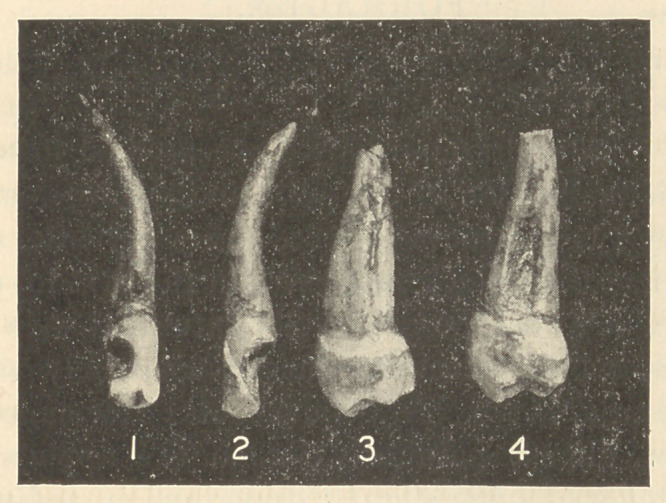# Studies in Evolution

**Published:** 1898-06

**Authors:** Eugene S. Talbot

**Affiliations:** Chicago, Ill.


### STUDIES IN EVOLUTION.

BY EUGENE S. TALBOT, M.D., D.D.S., CHICAGO, ILL.

Hereditary and atavistic specimens illustrating the evolution of man occur in dental practice, which are of great interest. "Recent discoveries," as Tomes remarks, "have proved that retrogression is a more common phenomenon in teeth than was formerly supposed." Hence, biology and comparative anatomy in relation to the jaws and teeth are studies of especial value to every dentist.

The degenerate patients of the dentist afford rare opportunities for such study. The illustration nicely demonstrates, from the dental stand-point, that man as a vertebrate animal sprang from the lower marsupials and lemurs, and that the bicuspids and molars are developed from single root and cone teeth. The illustration shows right and left lateral incisors (Figs. 1 and 2) and right and left second bicuspids (Figs. 3 and 4). The roots are usually long and are considerably curved, thus affording great strength, as in the carnivora. The roots of the bicuspids are composed of three and four smaller roots fused together with grooves showing their outline. The crowns are made up of five cusps each, showing the transition stage from a bicuspid to a molar tooth. The person from whose mouth these teeth were taken was a woman, forty-two years of age. She had a contracted chest, large hands and feet, and thyroid gland. The arms were unusually long, her jaws were large and well developed, face sunken, with high cheek bones, ears large, with right higher than left, right eye higher than left. That such an individual should possess atavistic teeth would seem to follow as a matter of course.

## Figures and Tables

**Figure f1:**